# Transcriptomic analysis of the effects exerted by curcumin on dihydrotestosterone-induced ovarian granulosa cells

**DOI:** 10.3389/fendo.2025.1522269

**Published:** 2025-02-13

**Authors:** Dejian Chen, Qian Yu, Shuhao Sheng, Lingshi Cai, Jisuo Zheng, Yaling Zhang

**Affiliations:** ^1^ School of Medicine, Jiaxing University, Jiaxing, China; ^2^ Department of Anesthesiology and Pain Research Center, The Affiliated Hospital of Jiaxing University, Jiaxing, China; ^3^ Pathology Department, Zhejiang Rongjun Hospital, Jiaxing, China

**Keywords:** hyperandrogen, granulosa cells, polycystic ovary syndrome, curcumin, RNA sequencing

## Abstract

**Purpose:**

Hyperandrogenism is a leading cause of developmental retardation in ovarian granulosa cells. Previous studies have indicated that curcumin significantly improves follicular dysplasia, a characteristic of the polycystic ovary syndrome. Our purpose was to explore the signaling pathways which enable curcumin to protect the development of hyperandrogen-induced granulosa cells.

**Methods:**

Ovarian granulosa cells treated with or without curcumin at different dihydrotestosterone (DHT) levels, were screened for cell viability, reactive oxygen species production, and apoptosis. RNA sequencing (transcriptome sequencing) was used to determine global gene expression in DHT-induced granulosa cells treated with curcumin.

**Results:**

24 hours of combined curcumin and DHT treatment inhibited granulosa cell viability in a dose-dependent manner. Curcumin upregulated estrogen synthesis-related enzymes, downregulated lipid metabolism-related genes and the glucuronic acid process, inhibited androgen receptor (AR) activity, significantly improved cell viability, and corrected granulosa cell development. Gene set enrichment and genome transcriptome pathway analyses revealed the potential role played by curcumin in protecting granulosa cell development.

**Conclusion:**

High androgen levels may disrupt steroid hormone synthesis and lipid metabolism pathways associated with granulosa cell development, thereby activating AR and inhibiting estrogen biosynthesis. Curcumin restores granulosa cell development by correcting abnormal steroid gene expression and disordered lipid fatty acid metabolism.

## Graphical Abstract

By Figdraw.

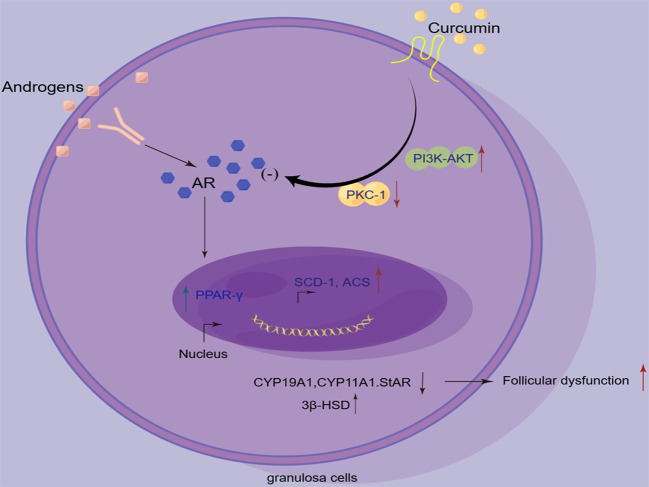

## 1 Introduction

The follicle is composed of the germ, granulosa, and follicular membrane stromal cells. The growth and development of granulosa cells are key to follicle development. Gonadotropins regulate the proliferation and differentiation of granulosa cells into granulosa cell groups which exhibit different degrees of differentiation. These granulosa cell groups regulate the growth and maturation of oocytes via paracrine and gap junction communication (1, 2).

Androgens exist in many biologically active forms, such as testosterone (T) and dihydrotestosterone (DHT). The enzyme, 5-α reductase converts T into DHT, which shows higher bioactivity and affinity for androgen receptors than T. Androgens activate androgen signaling by binding to androgen receptors (ARs), following which they enter the nucleus and interact with the cis-acting enhancer sequences of the androgen response (AR) element on target genes to regulate gene expression at the transcriptional level, thereby participating in a variety of important physiological functions. Ovarian granulosa cells express AR. The androgen signaling pathway regulates early follicle growth, development, and follicle atresia (3, 4). Studies have shown that both ovarian granulosa cell division and proliferation are inhibited in women with PCOS (5–8), while their apoptosis is significantly increased. In general, arrested granulosa cell development is closely associated with follicular dysplasia in PCOS, while a long-term continuous hyperandrogenic environment facilitates the development of granulosa cells. Thus, we investigated the effect of DHT on granulosa cells, with the intention of providing an experimental basis for further exploration of DHT-based regulation of follicular growth and development as well as the effects of androgens on the ovarian reserve.

Curcumin, which is a lipophilic polyphenolic compound extracted from the rhizomes of the Zingiberaceae family, easily permeates the cell membrane. Its molecular formula is C_21_H_20_O_6_, and its relative molecular weight is 368.38. It exhibits potent biological and pharmacological activities (9, 10). Many studies have treated PCOS with curcumin. Curcumin significantly reduces androgen levels, corrects sex hormone disorders, reduces waist-hip ratios, body mass indexes, triglycerides, total cholesterol contents, and ovarian indexes, and improves the estrous cycle, regulates blood glucose, and reduces inflammatory factors in patients with PCOS (11–14). In addition, a pervious study of ours indicated that *in vitro* treatment with curcumin significantly reduced ROS levels in DHT-induced granulosa cells, increased mitochondrial membrane potential, down-regulated the expression of *p-IRE1α* and *XBP1s* and their proteins, and inhibited the apoptosis of granulosa cells (15). However, another study reported that T induces only a slight increase in apoptosis rates and ovarian granulosa cell related signaling. Because DHT is a steroid hormone, and the production of T is induced by 5-α reductase, we speculated that the reason for apoptosis in granulosa cells induced by T being so insignificantly low may be attributed to the abnormal intracellular 5-α reductase levels seen in ovarian granulosa cells (16). The production of steroid hormones is regulated by many genes, including those encoding cytochrome P450 aromatase. Aromatase (CYP19) is a rate-limiting enzyme that converts androgens to estrogen during the final stage of steroid production. It is found in the ovaries, the placenta, and other tissues. A decrease in the expression or activity of CYP19A1 leads to increased levels of ovarian androgens. *In vitro* studies have shown that high androgen exposure inhibits the transcription of *CYP19A1* and *CYP11A1* in human ovarian granulosa cells (KGN), thereby affecting follicular development. However, few studies have investigated the direct effects of curcumin on primary granulosa cells, and those studies that did do so generally used KGN or animal models (17–19). In this study, we applied RNA sequencing to elucidate the genome-wide expression of primary granulosa cell development affected by high androgen levels, as well as the effects of curcumin on intracellular steroid hormone biosynthesis and lipid metabolism, and to explore the potential mechanisms underlying these processes.

Sequencing has revolutionized biology and medicine in recent years, providing single-base-level precision for high-throughput understanding of nucleic acid sequences (20). The current study used transcriptomic analysis to investigate the potential mechanism(s) underlying the effects of curcumin treatment on granulosa cells induced by DHT.

## 2 Materials and methods

### 2.1 Animals

Specific pathogen-free (SPF), 21day old Sprague Dawley^®^ (SD) female rats were obtained from the Shanghai Xipuer-Bikai Laboratory Animal Co., Ltd. (Shanghai, China). The animals were housed under conditions involving controlled standard temperatures (22 ± 2°C) and light (12:12 h, light: dark). All experiments were performed with the permission of the Institutional Animal Care and Use Committee (IACUC) and approved by the Institutional Research Animal Committee of Jiaxing University.

### 2.2 Chemical drugs

Curcumin, a natural polyphenol with a purity of 99%, was purchased from Sigma (Sigma-Aldrich, St. Louis, Massachusetts, USA). Curcumin powder was dissolved in DMSO to obtain a 50 mM reserve solution, which was then diluted into a 50 µM working solution using cell culture medium. A gradient concentration (5, 10, 15, 20, 25, 30, 50 µM) was utilized to detect the effect of curcumin on the viability of granulosa cells. Finally, optimal therapeutic concentrations were determined. Granulosa cells were treated with different concentrations of DHT (Selleck, Shanghai, China) (0.1, 0.2, 0.3, 0.4, 0.5 and 1 µM). Previous studies found that granulosa cells induced using 0.5 µM DHT exhibited the highest activity (4, 21).

### 2.3 Primary cell cultures and treatments

The rats were injected with pregnant maternal serum gonadotropin (PMSG) (20IU) 48 h in advance (22). All rats were euthanized via cervical dislocation. Following immersion in 75% alcohol and disinfection for 20 min, the ovaries were rapidly dissected on a clean bench under aseptic conditions and placed in pre-cooled sterile PBS to remove surrounding tissues and surface capsules. After washing with PBS, the ovaries were placed in pre-cooled DMEM/F12 (Gibco, USA). Subsequently, the follicles were punctured under an anatomical microscope with a 25-gauge needle to release granulosa cells into DMEM/F12 medium supplemented with 10% fetal bovine serum (Gibco, Grand Island, NY, USA) and 1% penicillin-streptomycin (Gibco). 200 mesh stainless steel cell sieve filtration. Centrifuged at 1000r/min for 5 min and washed, following which the supernatant was discarded, and the cells collected. A single-cell suspension was prepared by adding DMEM/F12 supplemented with 10% fetal bovine serum and 1% penicillin-streptomycin to the loose cell mass deposited at the bottom of a centrifuge tube. The cells were diluted to produce a 1.0 x 10^5^/mL cell suspension and inoculated in a culture flask. After 24 h of culture in a 5% CO_2_ incubator at 37°C, the culture medium was changed once to remove non-adherent cells.

The experimental groups were as follows: the control group (Control); the model group (DHT); and the curcumin treatment group (DHT + CUR). Treatment details were as follows: the control group (DMSO); the model group (treated with 0.5 μM DHT for 48 h); and the curcumin treatment group (treated with 0.5 μM DHT, gently mixed and then treated with CUR for 48 h).

### 2.4 CCK8 assay

Granulosa cells were seeded in 96-well plates at a density of 1 × 10^5^/mL and then treated with different concentrations of DHT (0, 0.1, 0.2, 0.3, 0.4, 0.5 and 1 μM) or a concentration gradient of curcumin (5, 10, 15, 20, 30 and 50 µM) for 24h. Then, granulosa cells were optimally induced by treating with DHT (0.5 μM), following which 20 μM curcumin was added for 48 h. A cell counting kit-8 (CCK-8) solution (10 μL; A311-02-AA, Vazyme Biotech, Nanjing, China) was added to each well, followed by incubation for 4 h at 37°C. The absorbance of each sample was measured at 450 nm using a microplate reader.

### 2.5 ELISA

Cell incubation supernatant was collected and its Anti-müllerian hormone (AMH) and Progesterone content were detected using the ELISA kit (Elabscience Biotechnology). The ELISA ranges were 62.5-4000 pg/mL and 0.31-20 ng/mL, and the sensitivities were 37.5pg/mL and 0.15 ng/mL, respectively. According to the manufacturer’s instructions, 450 nm was selected as the most suitable wavelength for measuring absorbance.

### 2.6 Immunofluorescence

Cell climbing tablets were fixed with 4% paraformaldehyde at 24°C for 30 min and blocked with 5% bovine serum albumin (Boster) for 30 min. Sections were then incubated overnight at 4°C with primary antibodies against 3β-HSD (1:200, sc-100466, SantaCruz, California, USA), StAR (1:200, 12225-1-AP, Proteintech, Chicago, USA), and AR (1:200, ab52615, Abcam, Cambridge, UK). The fluorescently labeled secondary antibodies were then incubated at 24°C for 2 h. Nuclei were counterstained with 4′,6-diamidino-2-phenylindole (C1002, Beyotime, Shanghai, China) at a dilution of 1:2000 for 30 min. Images were captured using an Olympus laser-scanning confocal microscope (FV3000; Tokyo, Japan).

### 2.7 RNA extraction and RT-PCR analysis

Total RNA was extracted from DHT- and curcumin-treated granulosa cells using an RNA Miniprep kit, as described by the manufacturer (Vazyme Biotech), while a reverse transcription kit (R223-01, Vazyme Biotech, China) was used for cDNA synthesis. The primer sequences are listed (**Table 1**) Quantitative RT-PCR was performed via an ABI ViiA 7 Real-Time PCR system (ABI, USA) using SYBR Green PCR Master Mix (Q441-02, Vazyme, China). Expression levels were calculated using the 2^-ΔΔCT^ method.

**Table 1:** Primers of the genes used in the study.

Genes		
3β-HSD	F	GTACATTTATGGGGAGAGAAG TCC
	R	CCAGGCCACATTGCCTACATA
CYP19A1	F	AACCCGAGCCTTTGGAGAA
	R	GGCCCGTCAGAGCTTTCA
CYP11A1	F	GGATGCGTCGATACTCTTCTCA
	R	GGACGATTCGGTCTTTCTTCCA
StAR	F	CCCCGTGACTTTGTGAGC
	R	CGTAAGTTTGGTCTTAGAGGGA
AKR1C3	F	GGGATCTCAACGAGACAAACG
	R	AAAGGACTGGGTCCTCCAAGA
UGT2B4	F	TCGAGAGACTTAGACACAAG
	R	CCAATAGGATCTAATAAGCC
PKC	F	GCTGCTGGGGAGTTTACTGG
	R	GATGCCATCTGTTCTCCCGTG
Cyclin D1	F	AGAGGCGGATGAGAACAAGC
	R	CCTTGTTTAGCCAGAGGCCG
ACS	F	GTCACAGAAGTGGACGGAGTG
	R	CAAATTAACCATATACCCGTCGA
SCD-1	F	CCTTATGTCTCTGCTACACTTGGG
	R	ATGAGCTCCTGCTGTTATGCC
β-Actin	F	GTGCTATGTTGCTCTAGACTTCG
	R	ATGCCACAGGATTCCATACC

### 2.8 RNA- seq data analysis

An NGS analysis was performed on a BGISEQ- 500 platform by oeBiotech Genomic Services, generating, on average, approximately 6.86G reads per sample. After filtering, clean HISAT reads were mapped to the reference genome. On average, 90.73% of reads were mapped, and clean reads were mapped to reference transcripts using Bowtie2 (v2.2.5); then the level of gene expression for each sample was measured via RSEM (v1.2.12). Next, we used Interferome 2.0, and EnrichR to annotate transcripts; clustered image maps (CIMs) were rendered using CIMminer. NGS data were deposited in the NCBI for Biotechnology Information Gene Expression Omnibus database. Sequence outcomes for each transcript were obtained as FPKM (fragments per kilobase of exons per million reads).

### 2.9 Statistical analysis

Experiments were performed in a blinded and randomized manner. All data were expressed as mean ± SD. Differences between mean values were determined using the t-test in GraphPad Prism 7. Statistical significance was set at *p*<0.05.

## 3 Results

### 3.1 Effect of curcumin on DHT-induced granulosa cells viability

**Figure 1:**
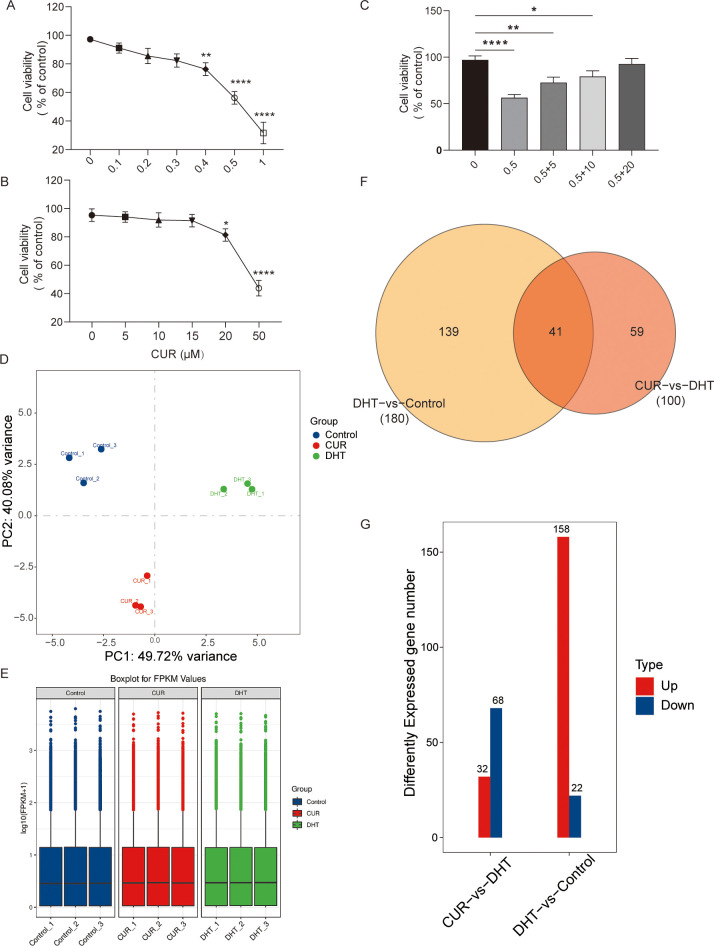
The effects of curcumin on the cellular viability and the transcriptome profile of granulosa cells. Primary granulosa cells were treated with curcumin with or without DHT. **(A–C)** Cell viability of granulosa cells treated with tunicamycin was analyzed using CCK-8 kits. **(D–G)** The results showed that 20µM curcumin may significantly increase DHT-induced decrease in cell viability. Data are presented as mean ± SD; ^*^ *p* < 0.05, ^**^ *p* < 0.01, ^****^ *p* < 0.0001. **(D-G)** Transcriptome profiles of curcumin-treated granulosa cells were verified via Cluster analysis, Box-whisker Plot, and differential gene distribution.

The concentration ranges of DHT (0, 0.1, 0.2, 0.3, 0.4, 0.5 and 1 μM) and curcumin (5, 10, 15, 20, 25, 30 and 50µM) were set according to a previous experiment (15, 23). We detected granulose cell viability, the cells were treated with Curcumin with or without DHT for 24 h via the CCK-8 method (10 μL; A311-02-AA, Vazyme Biotech, Nanjing, China). The results showed that the inhibitory effect of DHT on cell viability increased with increasing DHT concentration. The optimal concentration of curcumin which rescued DHT-induced decline in granulosa cell activity was 20µM (**Figures 1A–C**).

### 3.2 General analysis of comparative transcriptome

To determine the effects of curcumin on granulosa cells, we performed RNA sequencing of curcumin- and DHT-treated primary granulosa cells. Principal component analysis (PCA) revealed that the samples from DHT-induced granulosa cells clustered separately from those from untreated granulosa cells as well as those from curcumin-and DHT co-processed granulosa cells. Differential expression analysis was performed to identify changes. First, we compared the gene expression profiles of the control group with those of DHT alone group, and then compared those of the DHT alone group with those of the DHT-curcumin group, including the number of upregulated and downregulated genes. The transcriptome profiles of DHT-induced granulosa cells treated with curcumin were verified via cluster analysis, box-whisker plots, and differential gene distribution (**Figures 1D–G**).

### 3.3 Transcriptomic profile of DHT-induced granulosa cells vs Control

**Figure 2:**
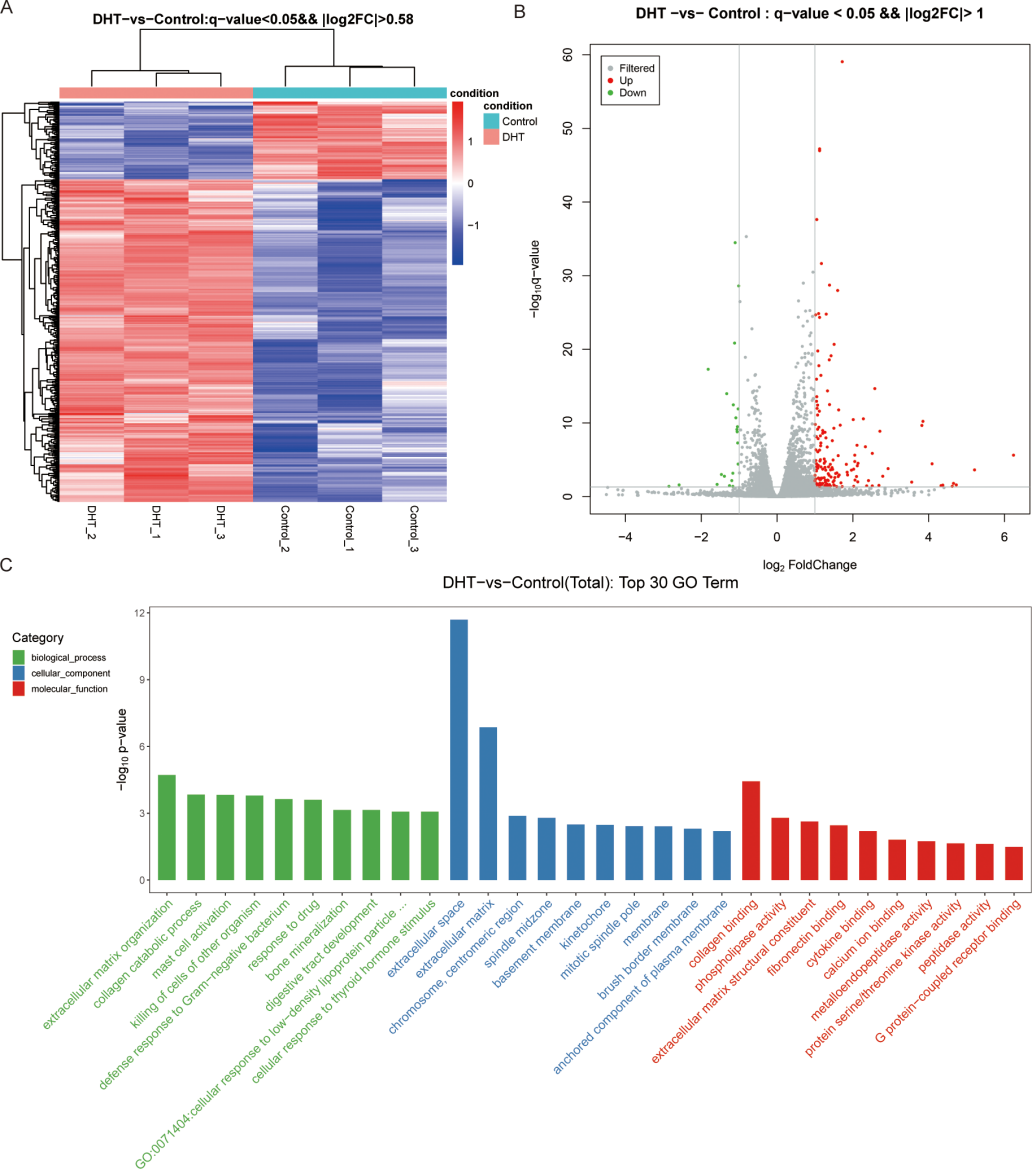
High-throughput RNA sequencing enrichment analysis of DEGs in granulosa cells treated with or without DHT. **(A)** Heat map showing the relative transcript levels of differential genes in granulosa cells showing expression pattern changes. **(B)** Volcano plot showing considerably downregulated (green dots) and upregulated (red dots) genes. **(C)** DHT vs Control, the top30 GO enrichment analysis entries (GO entries with more than 2 corresponding differential genes in the three classifications were screened, and 10 entries were ranked from largest to smallest according to the -log10p-value corresponding to each entry), including biological process, cellular component and molecular functions, are shown in the bar chart.

**Figure 3:**
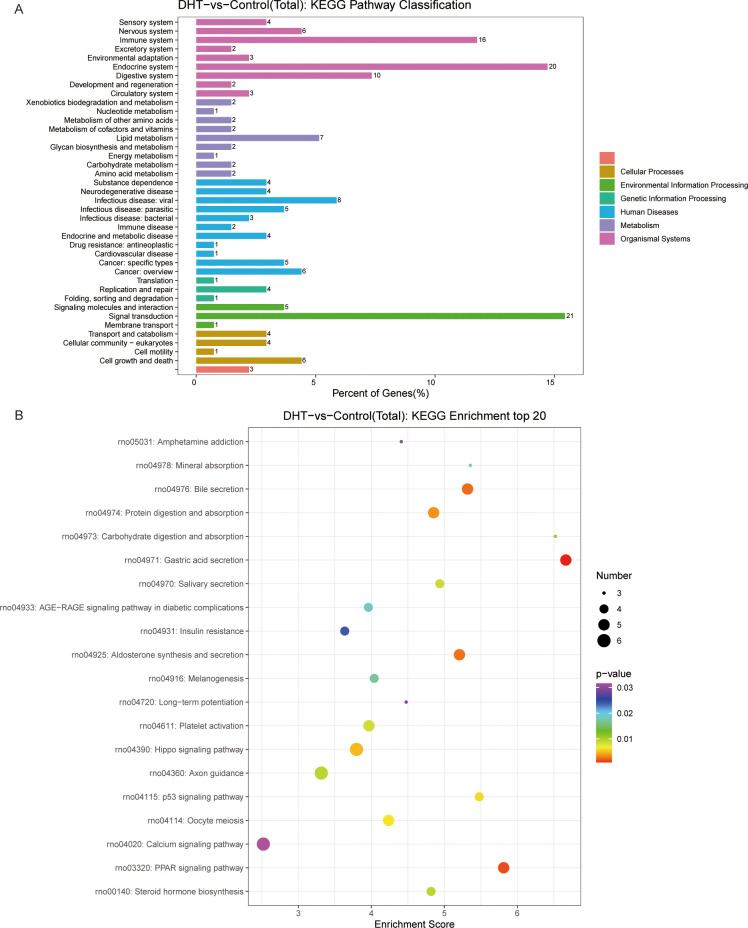
KEGG pathway enrichment analysis of significant DEGs and the top20 KEGG enriched bubbles. **(A)** DHT vs Control KEGG pathway enrichment analysis of significant DEGs. **(B)** KEGG enriched top20 bubbles. In the figure, horizontal axis represents the enrichment score. Entries with larger bubbles contain more different-protein coding genes with bubble color varying from purple-blue-green-red. The smaller the enrichment p-value, the greater the significance. Compared with those in the control group, the pathways enriched in differentially expressed genes in the model group were gastric acid secretion and PPAR signaling pathway.

In total, 500 DEGs were identified in DHT-induced granulosa cells, of which 403 were upregulated and 97 were downregulated in the heat map (**Figure 2A**). Next, we performed volcano plot analysis for all DEGs, which showed 158 upregulated and 22 downregulated genes (**Figure 2B**). Gene Ontology (GO) enrichment analysis was used to classify the functions of these DEGs, resulting in them being classified into three categories: biological processes; cellular components; and molecular functions (**Figure 2C**). KEGG pathway enrichment analysis was used to identify the DEGs involved in major biochemical and signal transduction pathways. Enrichment analysis of DEGs in DHT-induced group versus control group was performed using the KEGG database (**Figure 3A**). This indicated that the DEGs were enriched in gastric acid secretion (rno04971), the PPAR signaling pathway (rno03320), bile secretion (rno04976), aldosterone synthesis and secretion (rno04925), and protein digestion and absorption (rno04974), as well as the Hippo signaling pathway (rno04390) and several other pathway categories (**Figure 3B**).

### 3.4 Transcriptome profile alteration under curcumin treatment

**Figure 4:**
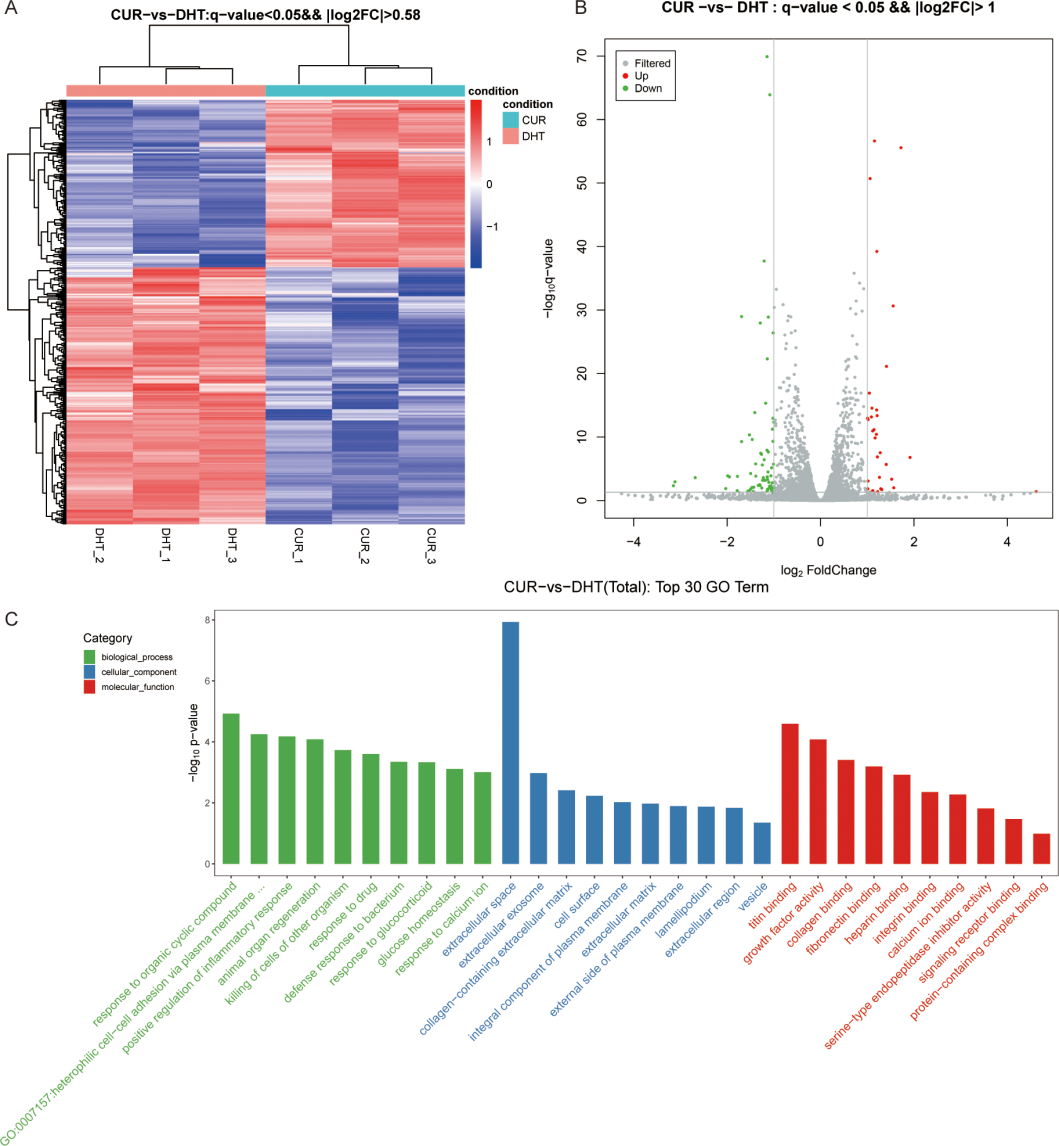
High-throughput RNA sequencing enrichment analysis of DEGs in granulosa cells treated with DHT and curcumin. **(A)** Heat map showing the relative transcript levels of the differential genes in granulosa cells showing changes in expression patterns. **(B)** Volcano plot showing considerably downregulated (green dots) and up-regulated (red dots) genes. **(C)** CUR vs DHT, the top30 GO enrichment analysis including biological process, cellular component and molecular functions, are shown in the bar chart.

**Figure 5:**
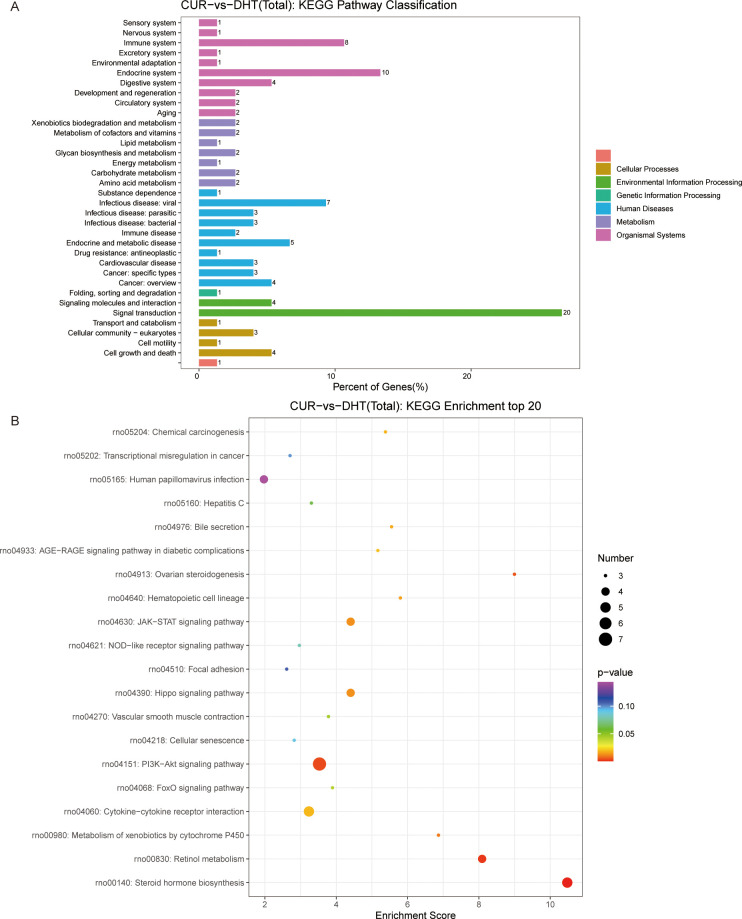
KEGG pathway enrichment analysis of significant DEGs and the top20 KEGG enriched bubbles. **(A)** CUR vs DHT, KEGG pathway enrichment analysis of significant DEGs. **(B)** Top20 KEGG enriched bubbles. Compared with those in the model group (DHT group), the pathways enriched in differentially expressed genes in the medication group (CUR group) were the PI3K-Akt signaling pathway and steroid hormone biosynthesis.

We investigated the protective effects exerted by curcumin on granulosa cells exposed to high androgen levels. We used transcriptomic profile analysis and identified 380 DEGs compared to the DHT-induced treatment, where these included 151 upregulated and 230 downregulated genes (**Figure 4A**). Volcano plot analysis of all screened DEGs showed 32 upregulated and 68 downregulated genes (**Figure 4B**). As mentioned above, the DEGs were classified into three categories: biological processes; cellular components; and molecular functions (**Figure 4C**). Enrichment analysis of DEGs in curcumin- and DHT-treated granulosa cells was performed using the KEGG database (**Figure 5A**). These showed that PI3K-Akt signaling pathway (rno04153), steroid hormone biosynthesis (rno00140), retinol metabolism (rno00830), JAK-STAT signaling pathway (rno04630) and Hippo signaling pathway (rno04390) and several other pathway categories were enriched in DEGs (**Figure 5B**). These enrichment pathways were highly similar to the pharmacological mechanisms of curcumin (rno00140). In addition, DEG enrichment was associated with steroid hormone biosynthesis, suggesting that curcumin treatment may correct the abnormal expression of steroid hormone-related genes in granulosa cells.

### 3.5 Changes in genes associated with classical granulosa cells developmental functions involving steroid hormones

**Figure 6:**
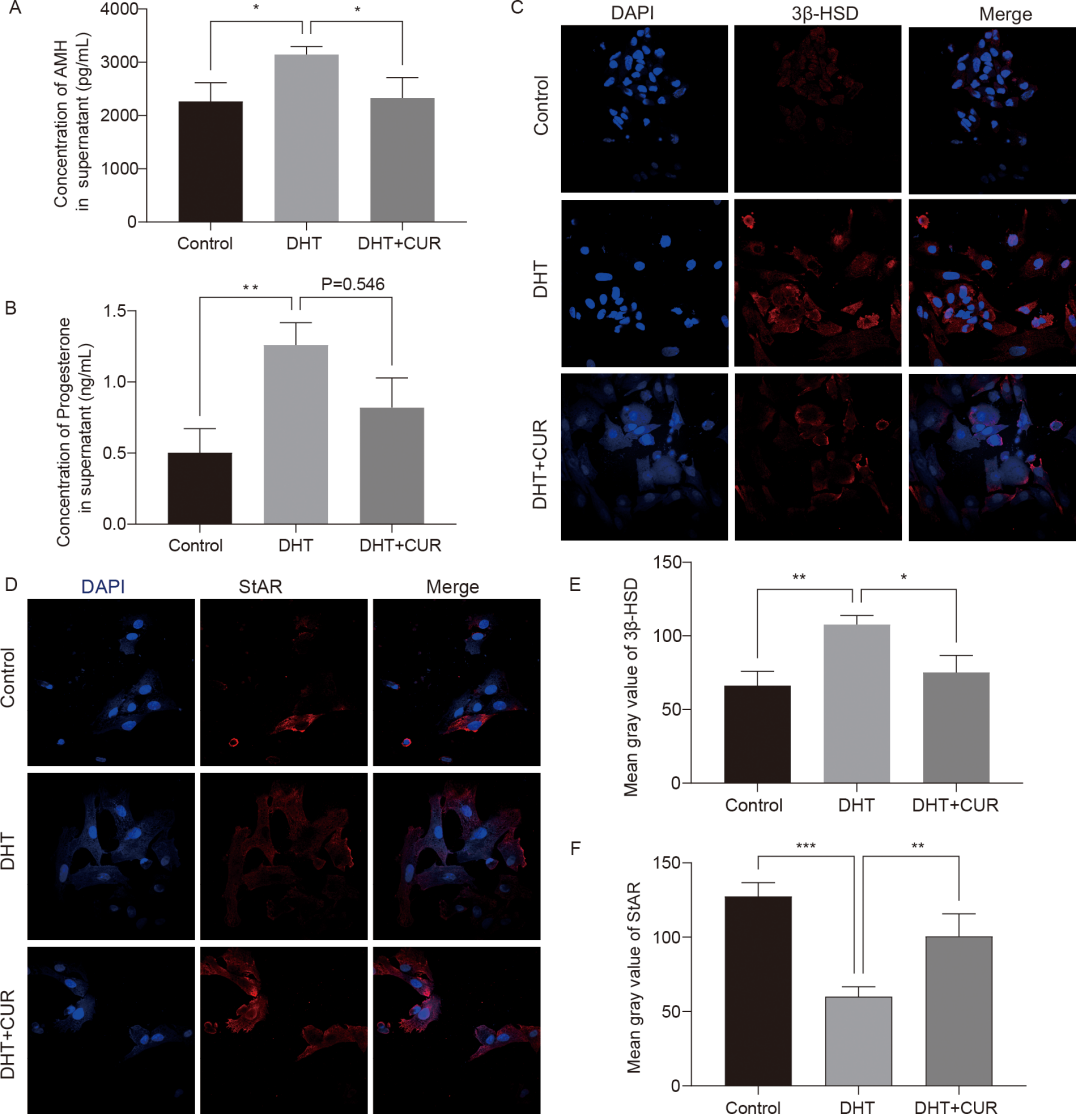
Expression levels of steroid hormone related factors changed by curcumin-related effects in granulosa cells. **(A, B)** AMH and Progesterone production in the cell incubation supernatant were measured. **(C–F)** The expression of 3β-HSD and StAR were detected and quantified by immunofluorescence. Results are presented as mean ± SD; ^*^ *p* < 0.05, ^**^ *p* < 0.01, ^***^ *p* < 0.001.

**Figure 7:**
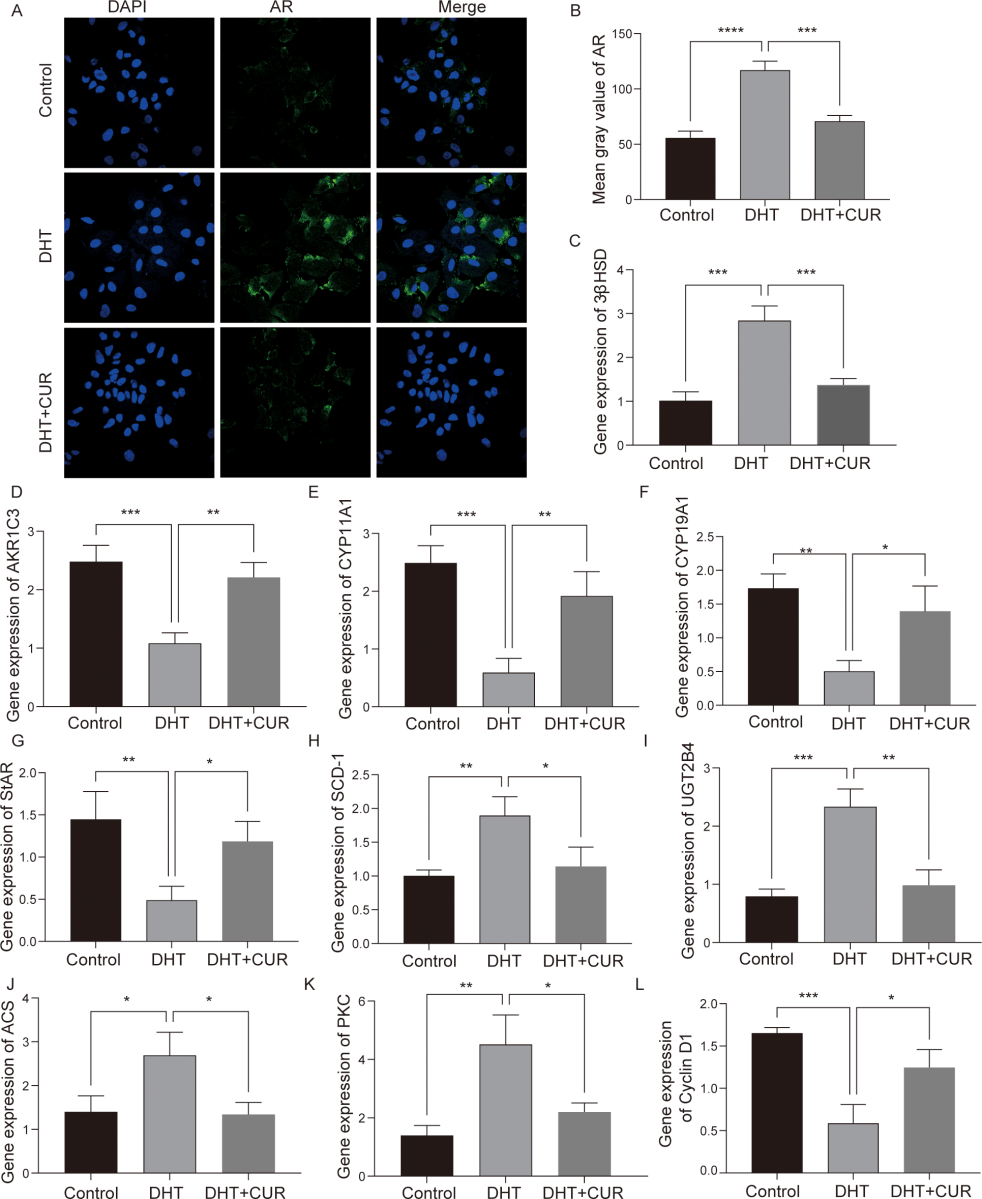
The expression of steroid-related genes, lipid fatty acid metabolism and cell cycle related factors after curcumin intervention in granulosa cells. **(A, B)** The expression of AR was detected and quantified via immunofluorescence. **(C–L)** mRNA was extracted from granulosa cells and the expression levels of *3β-HSD*, *AKR1C3*, *CYP11A1*, *CYP19A1*, *StAR*, *SCD-1*, *UGT2B4*, *ACS*, *PKC* and *Cyclin D1* were detected via RT-qPCR. Data are expressed as the mean ± SD; ^*^ *p* < 0.05, ^**^ *p* < 0.01, ^***^ *p* < 0.001, ^****^ *p* < 0.0001.

AMH expression is limited to gonads and secreted by granulosa cells of presinus and sinusoid follicles. AMH reduces the sensitivity of follicles to FSH and thereby inhibits follicular growth. The occurrence and development of PCOS are closely associated with AMH levels (24, 25). In this study, the ELISA kit were used to detect AMH and Progesterone secretion in DHT-induced granulosa cells treated with curcumin (the optimal treatment was 20 μM), while an immunofluorescence assay was used to detect the expression of 3β-HSD and StAR. The results showed that curcumin significantly decreased AMH and Progesterone secretion by DHT-induced granulosa cells (**Figures 6A, B**); 3β-HSD level was also reduced, and StAR level was significantly increased (**Figures 6C–F**). The differences were statistically significant. In addition, we verified whether curcumin affects AR activity in granulosa cells. Immunofluorescence assays revealed that curcumin had significantly inhibited AR expression (**Figures 7A, B**).

### 3.6 Validation of DEGs and signaling pathway activation in curcumin and DHT treated granulosa cells

To verify transcriptome data, we used by Q-PCR to further analyze the DEGs and the activation of signal transduction pathways in control, DHT- and curcumin-treated macrophages. We performed validation against 9 of the overlapping genes and found that the results were consistent with RNA-Seq results (**Figures 7C–L**). CYP11A1, CYP11A1 and StAR mRNA levels were most significantly downregulated, while UGT2B4, 3β-HSD and PKC mRNA levels were upregulated. In summary, the transcriptomic data provided further clues regarding the effects exerted by curcumin on steroid metabolism and granulosa cell function.

## 4 Discussion

Cytokines secreted by granulosa cells participate in the selection of dominant follicles as well as the regulation of follicle atresia, via complex signal transduction (26, 27). FSH receptors as well as LH receptors, present on the surface of granulosa cells, specifically express 17β-hydroxysteroid dehydrogenase-1 and cytochrome P450 (P450arom) aromatase. During the later stages of follicle development, granule cells catalyze the conversion of androstenedione (A4) and T to estrone (E1) and estradiol (E2) under the action of P450 aromatase, thereby promoting follicle development and dominant follicle formation (28–30). However, excessive androgens in women affect the balance between androgens, AMH, and the follicle-stimulating hormone (FSH), which in turn enhances FHS activity in granulosa cells, promotes AMH expression in presinus follicles and small antral follicles, and inhibits granulosa cell proliferation and follicle maturation, leading to follicular developmental disorders (31). In this study, we found that the AMH level of the supernatant in the DHT-induced model group was significantly increased compared to that in the control group. In addition to affecting follicular development, excessive androgen levels may lead to metabolic disorders (32). We used RNA sequencing to compare the transcriptional responses of primary cultured granulosa cells following hyperandrogen induction and curcumin treatment. We identified the DHT-mediated transcriptional signatures of granulosa cells with DEGs to understand the pathogenesis of hyperandrogen induction, which may help prevent PCOS. The results of this experiment showed that compared with hyperandrogen-induced granulosa cells, primary follicular granulosa cells in the normal control group showed 180 different genes, 38 types of refining functions, and 20 significantly different signaling pathways, including steroid hormone synthesis, lipid metabolism, cell proliferation, and energy metabolism. To verify the sequencing results, we screened five genes associated with follicular development. Compared with those of control primary granulosa cells, *CYP11A1, CYP19A1*, *StAR* and *AKR1C3* were downregulated, while *AR, 3β-HSD* and *UGT2B4* genes were upregulated in DHT-induced dysplastic granulosa cells. These results may provide a theoretical basis for further analyses of molecular mechanisms underlying GD-induced follicular atresia caused by granulosa cell dysplasia.

Previous studies of ours revealed that hyperandrogens induces significant follicular fibrosis in the ovaries of PCOS-like rats and that such fibrosis is associated with mitochondrial damage and oxidative stress (OS) in follicular granule cells. Hyperandrogenism activates the NLRP3 inflammasome in granulosa cells leading to pyroptosis (4, 22). Furthermore, endoplasmic reticulum stress, induced by hyperandrogens resulting in the apoptosis of granulosa cells via the IRE1α-XBP1 pathway, plays an important role in the PCOS-related follicle development disorder (15, 23). Therefore, identification of factors and interventions that affect the functioning of granulosa cells may help elucidate the pathogenesis of PCOS. In this study, granulosa cells were co-treated with curcumin and DHT for 24 h. DHT inhibited granulosa cell viability in a dose-dependent manner and also increased apoptosis. Similarly, we have previously reported that DHT treatment reduced the viability of rat primary follicular granulosa cells (33). However, another study reported that T had induced a slight, and not significant, increase in the rate of apoptosis of ovarian granulosa cells (16). This may be attributed to the complex effects of abnormal granulosa cell development in androgen-rich environments. Some reports have indicated that gastric parietal cells synthesize and secrete large amounts of estrogen into the portal vein of both male and female rats (34, 35). However, the detailed functioning of gastric estrogen remains unclear. Thus, further studies aimed at elucidating the role of gastric estrogens in the liver and ovaries, the regulatory factors modulating gastric estrogens, the association between E2 and PCOS, and the feasibility of clinical applications are required. Studies have shown that pioglitazone significantly improves lipid droplet deposition, triglyceride (TG) and total cholesterol (TC) levels, and liver weight in PCOS-IR rats (36, 37). These findings may shed light on substances that may be useful as potential targets in the treatment of PCOS associated with metabolic disorders.

The top 20 bubbles enriched by KEGG showed that, compared to the control group, the pathways in the DHT group that were enriched in DEGS were mainly gastric acid secretion and the peroxisome proliferators-activated receptors (PPARs) signaling pathways. PPARs are members of the nuclear receptor superfamily. PPAR agonists improve insulin sensitivity in several ways, including the regulation of glycolipid metabolism and anti-inflammatory effects, and indirect improvement of OS states. PPARs, expressed in the ovaries, plays an important role in the female reproductive tract and affects fertility. Most studies have reported the expression levels of PPARγ (and PGC-1α) in women with PCOS. Although some studies have shown the hypothesis that there is no difference in PPARγ expression between PCOS patients and controls, certain researchers reported significant upregulation, whereas several study teams suggested significantly lower expression levels (38). It has been reported that AMPK/PPAR-γ/SIRT1 pathway is involved in the anti-inflammatory and antioxidant capacity of granulosa cells (39). Study indicates that PPAR-α may have an inhibitory effect on DHEA-induced ferroptosis in granulosa cells (40). So far, natural medicines have been reported to have an effect on PPAR expression in PCOS. Arandomized, double-blind, placebo-controlled trial to evaluate the effect of curcumin in women suffering from PCOS and revealed PPARγ upregulation after curcumin administration (41). Analogously, Heshmati et al. carried out a randomized placebo-controlled clinical trial and treated 36 PCOS patients with the biologically active phytochemical ingredient curcumin, which significantly increased gene expression of PGC-1a (13). A study demonstrates that curcumin effectively restores ovarian morphology, hormone levels, and estrous cycles in PCOS rats. These effects may be linked to its ability to reduce oxidative stress in ovaries via the upregulation of PPAR-γ (42). In conclusion, further and more consistent studies on the role of PPAR in PCOS are still needed.

Curcumin is a free radical scavenger, as well as a reducing agent and an inhibitor of DNA damage (43, 44). Curcumin treatment reduces the ovarian index as well as the number of primordial follicles and the corpus luteum, alleviates disorders of the estrus cycle and sex hormone levels, regulates blood sugar levels, and reduces the expression of inflammatory factors in serum and tissues (45). OS is a leading cause of apoptosis and follicular atresia in granulosa cells (46). In follicles, the immune system plays an important role as an antioxidant. Curcumin is an important antioxidant and apoptotic compound. Based on a previous study, we treated DHT-induced primary granulosa cells with the optimal therapeutic concentration of curcumin (15, 23). RNA sequencing and KEGG enrichment analysis revealed significantly different pathways including the PI3K-Akt signaling pathway, steroid hormone biosynthesis, retinol metabolism, JAK/STAT signaling pathway and cytokine receptor interaction. The PI3K-Akt mediated pathway plays an important role in regulating cell proliferation and survival as well as the balance between primordial follicle stasis and follicle-activated development in the ovary (47); enzymes that catalyze steroid hormone biosynthesis are closely associated with ovarian function and granulosa cell development. This pathway, which includes functional genes encoding anabolic steroids, such as CYP19A1, CYP11A1, StAR and 3β-HSD, directly or indirectly participates in the synthesis of ovarian steroid hormones and AMH. The JAK/STAT pathway represents a major mechanism utilized by cytokines and growth factors for signaling. Studies have reported that both JAK1/3 and STAT1/3 are expressed in granulosa cells, suggesting that the protective effects exerted by curcumin on granulosa cell development may be mediated via the JAK/STAT pathway (48, 49). Previous studies have reported that polyphenols and alkaloids are multitarget natural products that may be used to exert neuroprotective effects via the modulation of JAK/STAT and IRS/PI3K signaling pathways (50). However, only a few studies have investigated the possibility of using curcumin to target the JAK/STAT pathway as a means of regulating granulosa cells and follicle development. Therefore, further investigations into the role played by this pathway in the functioning of follicular granulosa cells appear to be warranted. Curcumin maybe could represent great therapeutic alternatives for patients who suffer from severe PCOS symptoms but remain skeptical about standard hormonal therapy.

In conclusion, we studied the protective effects exerted by curcumin on DHT-induced primary granulosa cells using transcriptomic analysis. We utilized RNA sequencing to identify potentially relevant genes (*CYP11A1, CYP19A1, StAR, AKR1C3, 3-β-HSD*, and *UGT2B4*) as well as pathways of action involved in steroid hormone synthesis and granulosa cell functioning, such as the PPAR signaling pathway and the PI3K/AKT signaling pathway. These findings not only explain the effects exerted by high androgen DHT on the developmental function of primary granulosa cells, but also the mechanism possibly underlying it. Moreover, our findings may provide a theoretical basis for the process by which curcumin protects follicular granulosa cells and reveal possible pathways associated with such a process.

## Data Availability

All relevant data is contained within the article: The original contributions presented in the study are included in the article material, further inquiries can be directed to the corresponding author.
